# Prevalence and drivers of HIV stigma among health providers in urban India: implications for interventions

**DOI:** 10.7448/IAS.16.3.18717

**Published:** 2013-11-13

**Authors:** Maria L Ekstrand, Jayashree Ramakrishna, Shalini Bharat, Elsa Heylen

**Affiliations:** 1 Center for AIDS Prevention Studies, University of California, San Francisco, CA, USA; 2 St. John's Research Institute, Bengaluru, Karnataka, India; 3 School of Public Health, University of California, Berkeley, CA, USA; 4 Department of Health Education, National Institute of Mental Health and Neurosciences, Bengaluru, Karnataka, India; 5 Centre for Health and Social Sciences, School of Health Systems Studies, Tata Institute of Social Sciences, Mumbai, Maharastra, India

**Keywords:** HIV stigma, stigma drivers, healthcare workers, India

## Abstract

**Introduction:**

HIV stigma inflicts hardship and suffering on people living with HIV (PLHIV) and interferes with both prevention and treatment efforts. Health professionals are often named by PLHIV as an important source of stigma. This study was designed to examine rates and drivers of stigma and discrimination among doctors, nurses and ward staff in different urban healthcare settings in high HIV prevalence states in India.

**Methods:**

This cross-sectional study enrolled 305 doctors, 369 nurses and 346 ward staff in both governmental and non-governmental healthcare settings in Mumbai and Bengaluru, India. The approximately one-hour long interviews focused on knowledge related to HIV transmission, personal and professional experiences with PLHIV, instrumental and symbolic stigma, endorsement of coercive policies, and intent to discriminate in professional and personal situations that involve high and low risk of fluid exposure.

**Results:**

High levels of stigma were reported by all groups. This included a willingness to prohibit female PLHIV from having children (55 to 80%), endorsement of mandatory testing for female sex workers (94 to 97%) and surgery patients (90 to 99%), and stating that people who acquired HIV through sex or drugs “got what they deserved” (50 to 83%). In addition, 89% of doctors, 88% of nurses and 73% of ward staff stated that they would discriminate against PLHIV in professional situations that involved high likelihood of fluid exposure, and 57% doctors, 40% nurses and 71% ward staff stated that they would do so in low-risk situations as well. Significant and modifiable drivers of stigma and discrimination included having less frequent contact with PLHIV, and a greater number of transmission misconceptions, blame, instrumental and symbolic stigma. Participants in all three groups reported high rates of endorsement of coercive measures and intent to discriminate against PLHIV. Stigma and discrimination were associated with multiple modifiable drivers, which are consistent with previous research, and which need to be targeted in future interventions.

**Conclusions:**

Stigma reduction intervention programmes targeting healthcare providers in urban India need to address fear of transmission, improve universal precaution skills, and involve PLHIV at all stages of the intervention to reduce symbolic stigma and ensure that relevant patient interaction skills are taught.

## Introduction

Across cultures, HIV stigma has repeatedly been shown to inflict hardship and suffering on people living with HIV (PLHIV) [1], as well as to interfere with their decisions to seek HIV counselling and testing [2, 3], prevention of mother-to-child transmission (PMTCT) [4–8], and their willingness to disclose their infection to their children [9] or partners [10–13], which can in turn increase the likelihood of sexual risk taking. HIV stigma has also been found to be a barrier to participation in vaccine research [14] and to deter infected individuals from seeking timely medical treatment [15–17].

These findings have been reported in both resource-rich and resource-constrained settings. Even when treatment is sought, stigma fears can prevent individuals from following their medical regimen, which can lead to virologic failure and the development and transmission of a drug-resistant virus [18–21]. PLHIV in Senegal and Indonesia have reported avoiding or delaying treatment seeking for STI/HIV infections, out of fear of public humiliation and fear of discrimination by healthcare workers [22, 23]. Similarly, HIV stigma in Botswana, South Africa, Jamaica and India has been associated with delays in testing and treatment services, sometimes resulting in presentation beyond the point of optimal drug intervention [24, 25].

Unfortunately, health professionals are often named as one of the most important sources of stigma for PLHIV. In sub-Saharan Africa, studies have documented discriminatory practices, including patient neglect, provision of differential treatment based on HIV status, denial of care, breach of confidentiality, isolation and verbal abuse by healthcare staff [26–28]. High rates of refusal of care have also been reported among nurses in Jordan [29] and stigma and discrimination have been documented in some healthcare settings in India also [15, 30–37].

In the general population of healthcare seeking individuals, behavioural manifestations of HIV stigma appear to be driven by both fear of casual transmission (*instrumental stigma*) and pre-existing prejudice towards vulnerable groups (*symbolic stigma*) [38]. No such data are available for healthcare providers. Studies of caregivers in other resource-constrained settings suggest that unwillingness to care for PLHIV is associated with negative views, high caregiver burden, low knowledge levels and education, and having a higher income [39, 40]. Understanding the specific drivers of stigma and its effects on behaviour in each setting is vital to the development of effective stigma reduction interventions for a given population [41]. The parent study was designed to meet this need by examining levels of stigma and discrimination as well as their potential drivers among healthcare providers, patients and the general outpatient population in two large urban settings in India. Our previous papers report on stigma among PLHIV [13, 42, 43] and the general patient population [38]. This article analyzes data from the healthcare providers and is the only one to date in India, which directly examines stigma domains and their drivers in these three healthcare provider groups, allowing us to conduct inter-staff comparisons. Another unique strength of this study is that it is the only one that includes all types of hospitals (including charity, trust, non-profit, for profit, and public) available in India. It is also the only study that includes data from two high prevalence areas in India, which allows for some generalization of results.

## Methods

### Participants

The participants for this cross-sectional study were recruited in 2009 from different types of healthcare settings in Bengaluru and Mumbai, two large Indian cities located in the “HIV high prevalence” states, Maharashtra and Karnataka [44]. At the time of the study, this label was given by the Indian National AIDS Control Organization to any state reporting >5% HIV prevalence in at least one key population or >1% in antenatal clinic settings. Field sites included medical colleges, government hospitals or private facilities, both for-profit and not-for-profit. Participants’ professional experience with HIV patients ranged from none to extensive. To meet the inclusion criteria, potential participants had to have worked as a doctor, nurse or ward staff in the selected hospitals/clinics for at least six months; have direct patient contact; be at least 18 years of age; able to speak one of the study languages; and able to give informed consent. The term “ward staff” included anyone who worked on the ward at a lower level than a nurse and who had substantial patient contact (including washing, transporting, changing linens). Most ward staff have minimal education or training and typically assist nurses or doctors with medical interventions. They are also a source of information and serve as confidants to patients. Final numbers recruited in Bengaluru were 149 doctors, 195 nurses and 176 ward staff; for Mumbai the numbers were 156, 174 and 170, respectively.

### Procedures

For each institution, we initially approached the Hospital Superintendents or Medical Directors for permission and subsequently contacted the Department heads for assistance in recruiting nurses and ward staff. Doctors preferred that we contact them directly to set up individual appointments. Following recruitment, potential participants were administered informed consent by study staff and following consent, an interview was scheduled. Interviews were conducted face to face in the participant's preferred language (Marathi, Hindi or English in Mumbai; Kannada, Tamil or English in Bengaluru) by trained study staff in a private space at the work site, and lasted approximately one hour. Participants received an in-kind gift worth 250 rupees (about 5 USD) following their interview, consisting of packets of fancy nuts and dried fruits in Bengaluru and a shopping bag in Mumbai.

Study procedures were approved by the Institutional Review Boards of the University of California in San Francisco, the National Institute of Mental Health and Neurosciences in Bengaluru, the Tata Institute of Social Sciences in Mumbai and received clearance from the Indian government.

### Measures

The survey measures used in this study were based on research conducted by Herek [45–49] as well as on the theoretical model subsequently developed by our team [13]. Stigma scales and drivers found to be significantly associated with mental health outcomes and delay of care seeking in our previous research with PLHIV [13, 42] and with the general outpatient population [38] were included in these analyses.

#### Demographic information

All participants were asked about their gender, age, marital status, religion, education and HIV training.

#### Potential drivers of stigma

##### Contact with PLHIV

Participants indicated the frequency of professional contact with PLHIV (0=never to 4=daily), and whether they personally had ever known any PLHIV (0=no; 1=yes).

##### Transmission misconceptions

Four items described forms of casual social contact through which HIV cannot be transmitted. For each item, participants indicated whether they thought HIV could be transmitted this way. The number of incorrect responses was summed. A higher score reflects more misconceptions.

##### Transmission knowledge

Participants were also asked if they thought HIV could be transmitted by direct exposure to several kinds of bodily fluids, or by activities such as unprotected sex with PLHIV. The number of correct answers to 15 such items was calculated, with higher scores reflecting better knowledge.

##### Instrumental stigma

Two individual items measured how worried participants were (0=not at all to 3=very worried) about getting HIV-infected (i) at work and (ii) outside of work.

##### Negative feelings towards PLHIV

Participants reported their feelings towards PLHIV on a scale from 0 (very negative) to 100 (very positive). To control for individual tendencies to assign high or low scores in general, we subtracted the rating for PLHIV from a similar rating of feelings towards men or women in general, so that higher anchored scores reflect more negative feelings towards PLHIV.

##### Blame

Participants indicated their agreement with the statement “People who got HIV/AIDS through sex or drug use have gotten what they deserve,” on a scale from 0 (strongly disagree) to 4 (strongly agree).

##### Symbolic stigma

Six items assessed how much participants’ moral/religious beliefs and feelings towards key populations influenced their opinions about HIV (0=not at all to 4=a great deal). An overall score was computed as the mean of all items. Higher scores express greater stigma. This scale had excellent reliability, with a Cronbach's *α* of 0.93 for doctors and 0.81 for both nurses and ward staff.

##### Perceived community stigma norms

Ten items assessed participants’ perceptions of the prevalence of HIV-stigmatizing attitudes in their community on a five-point scale [13]. Answers were averaged into one score, with higher numbers indicating more perceived community stigma. Cronbach's *α* ranged from 0.85 for doctors to 0.82 for nurses.

### Stigma manifestations

#### Intent to discriminate against PLHIV in professional situations

Participants were presented with two hypothetical work situations involving care for an HIV-positive patient. One situation posed virtually no risk of contact with bodily fluids. The second situation posed a greater risk of such contact. Response options were dichotomized as stigmatizing (refusing or performing the task only with unnecessary precautions) versus non-stigmatizing (performing the task as they would with any other patient).

#### Intent to discriminate against PLHIV in non-professional contexts

This was assessed by two hypothetical situations: (1) having a child who attends a school with an HIV-positive student and (2) getting medical care at a clinic that treated PLHIV. Leaving the school/clinic or avoiding contact/demanding special precautions was scored as stigmatizing. In addition, participants expressed their agreement (0=strongly disagree to 4=strongly agree) with seven statements about avoiding social or personal contact with PLHIV. Stigmatizing responses were summed over the nine items, with higher scores indicating greater intent to discriminate.

#### Endorsement of coercive policies

Participants indicated their agreement (0=strongly disagree to 4=strongly agree) with 11 statements related to the rights of PLHIV to have a family, education, employment, and health care; the right to choose to disclose HIV status; and mandatory HIV testing. Items were dichotomized, and stigmatizing responses (strongly/somewhat agree) were summed. Higher scores reflect greater endorsement of coercive policies.

### Data analysis

Frequencies and summary statistics were used to describe participants’ responses in the three groups. Differences between the three healthcare worker types in categorical outcomes were assessed via Chi-square tests, and in continuous outcomes via analysis of variance, with Bonferroni post-hoc pairwise comparisons in case of a significant *F*-value.

Separate multiple regressions were performed for each type of healthcare worker, using endorsement of coercive policies, and intent to discriminate in personal and professional contexts as separate outcomes. Site (Bengaluru vs. Mumbai) was controlled for in all models. All predictors that were associated bivariately with an outcome at *p<*0.25 [50] were initially included in the model. In subsequent models, the variable with the largest *p*-value was removed until all remaining variables were significant at *p<*0.10. For endorsement of coercive policies and intent to discriminate in personal context, linear regressions were performed. The two items for intent to discriminate at work were modelled via separate logistic regressions. Model assumptions regarding homoscedasticity, multicollinearity and influential outliers were adequately met. The logistic regressions were performed using SAS 9.2., and all other analyses were performed using SPSS 18.0.2.

## Results

### Demographic characteristics

As can be seen in Table 1, approximately half of the doctors (46%) and ward staff (51%), and almost all of the nurses (98%) were female and most were married. The vast majority of doctors (86%) and ward staff (78%) were identified as Hindu, while 59% of the nurses reported being Hindu and 36% identified as Christians, which is common in Indian hospitals. The mean age was slightly higher among ward staff: 39, compared to 35 for nurses and 34 for doctors. Education level among ward staff varied from less than four years (32%) to more than 10 years (8%) of schooling, with 45% having at least some secondary education. By definition, education was more uniform among doctors and nurses. Median household income was Rs. 40,000 (about $735) per month for doctors, Rs. 15,000 ($276) for nurses and Rs. 6000 ($110) for ward staff.

**Table 1:** Demographic characteristics

	Doctors (*n=*305)		Nurses (*n=*369)		Ward staff (*n=*346)	

	%	*n*	%	*n*	%	*n*
Site						
Bengaluru	49	149	53	195	51	176
Mumbai	51	156	47	174	49	170
Male	54	165	2	6	49	170
Religion						
Hindu	86	262	59	219	78	269
Muslim	5	16	1	5	1	5
Christian	2	6	36	132	6	21
Buddhist	3	10	3	12	15	51
Others	4	11	0	1	0	0
Marital status						
Currently married	58	177	68	251	77	267
Never married	41	125	29	105	8	29
Previously married	1	3	3	12	15	50
Education						
≤4 years					32	110
5–7 years					23	80
8–10			0	1	37	129
>10 years	100	305	100	368	8	27
Age: mean (SD)	33.7	(9.9)	34.9	(10.3)	39.4	(9.6)
Monthly household income in rupees: median (range)	40,000	(4000–900,000)	15,000	(2700–100,000)	6000	(400–50,000)

### HIV-related knowledge and experience

As can be seen in Table 2, approximately 70% of doctors and nurses indicated that they had received some form of HIV training, compared to 44% of ward staff (*p<*0.001). Despite their higher levels of HIV education, doctors and nurses did not score significantly higher on transmission knowledge than ward staff (*p=*0.18). The mean scores on the transmission knowledge index ranged from 11.4 out of 15 (ward staff) to 11.7 (doctors). However, the groups did differ in their mean number of casual transmission misconceptions, with the highest number occurring among ward staff (mean=0.8 out of 4), lower among nurses (mean=0.6), and lowest among doctors (mean=0.4). Female ward staff reported less professional contact with PLHIV than their male colleagues (mean=2.1 vs. 2.5, respectively, *p<*0.05) and were, on average, less knowledgeable about HIV transmission (mean knowledge score=11.1 vs. 11.8; mean misconceptions=1.0 vs. 0.6 for females and males, respectively, *p<*0.001). Similarly, female doctors had slightly more misconceptions than male doctors (females: mean=0.5 vs. males: mean=0.3, *p<*0.05). There were no other gender-related differences reported.

**Table 2:** Frequencies of reported stigma and other key model variables

	Doctors (*n=*305)		Nurses (*n=*369)		Ward staff (*n=*346)		
		
	%	*n*	%	*n*	%	*n*	*χ*^2^
Received HIV training	73	223	71	263	44	152	77.73***
Professional contact w/PLHIV							
Never	2	6	4	13	9	32	23.02***
Less than weekly	50	152	51	187	49	170	
Weekly	16	48	13	49	11	37	
Daily	32	95	32	119	31	107	
Know PLHIV personally	26	78	27	99	28	98	0.63
Instrumental stigma^a^							
Worried about infection *at work*	78	237	72	264	51	175	60.55***
Worried about infection *outside of work*	17	52	26	96	27	94	10.09**
Blame^b^	50	153	70	259	83	284	79.72***
	
	Mean	SD	Mean	SD	Mean	SD	*F*^d^
	
Negative feelings towards PLHIV, anchored (−100 to 100)^c^	4_A_	26	11_B_	31	13_B_	39	7.22***
Perceived community stigma norms (0–3)	2.2_A_	0.6	2.3_A_	0.5	2.5_B_	0.5	20.54***
Symbolic stigma score (0–4)	1.7_A_	1.4	2.3_B_	1.1	2.2_B_	1.1	20.27***
Transmission misconceptions (0–4)	0.35_A_	0.76	0.56_B_	0.80	0.78_C_	1.08	18.10***
Transmission knowledge: items correct (0–15)	11.7_A_	1.6	11.5_A_	1.5	11.4_A_	1.8	1.73

a Proportion of participants answering “a little bit” to “very” worried.

b Proportion of participants who (strongly) agreed with the statement “People who got HIV from sex/drugs got what they deserved.”

c Anchored: PLHIV rating subtracted from own-gender rating, so scores<0 correspond to positive feelings, and scores>0 to negative feelings towards PLHIV.

d Means with different subscripts differ significantly (*p<*0.05) from each other (Bonferroni post-hoc pairwise comparisons).

† *p<*0.10

* *p<*0.05

** *p<*0.01

*** *p<*0.001.

More than 90% of all participants reported that they had experience caring for PLHIV, with about half in each group stating that they had at least weekly contact with HIV-positive patients. Just over a quarter of the participants in each group indicated that they had known a PLHIV personally. Nurses showed similar levels of worry about HIV infection at work as doctors, with about three quarters in both groups expressing such worries, compared to 51% of ward staff (*p<*0.001). Outside of work, more nurses (26%) and ward attendants (27%) reported worrying about HIV infection than doctors (17%, *p*<0.01).

### Attitudes towards PLHIV

Participants’ attitudes towards PLHIV, compared to their feelings towards men and women in general, differed significantly between the healthcare worker groups. Overall, doctors held the least negative feelings and ward staff the most negative, with the mean level of negative feelings towards PLHIV being 4 out of 100 (SD=26) for doctors, 11 (SD=31) for nurses and 13 (SD=39) for ward staff (*p<*0.001). A high proportion of participants in all three healthcare worker types agreed with the statement that people who acquired HIV through sex or drugs, “got what they deserved” – ranging from 50% of doctors, to 70% of nurses and 83% of ward attendants (*p*<0.001). The mean scores on the symbolic stigma scale were significantly lower for doctors (mean 1.7/4.0) than for nurses (2.3) and ward staff (2.2). Similarly, HIV-stigmatizing community norms were perceived to be higher by ward staff (mean=2.5/4.0) than by nurses (mean=2.3) and doctors (mean=2.2, *p<*0.001). There were significant gender differences with respect to feelings towards PLHIV among both doctors and ward staff. Female doctors reported significantly more negative feelings towards PLHIV (females: mean=8 vs. males: mean=0, *p<*0.01). Similarly, female ward staff held significantly more negative feelings towards PLHIV than their male colleagues (mean=19 vs. 8, respectively, *p<*0.05). In addition, female ward staff scored higher on perceived stigmatizing community norms (mean=2.6 vs. 2.4, respectively, *p<*0.001) and symbolic stigma (mean=2.3 vs. 2.1, respectively, *p<*0.05) than male ward staff. There were no other significant gender differences in any healthcare provider group with respect to attitudes towards PLHIV.

### Endorsement of coercive policies regarding PLHIV

Ward staff endorsed a mean of 6.7 out of 11 coercive policies, nurses endorsed on average 6.1 and doctors 4.8 (*p<*0.001). Mandatory testing for different groups was endorsed by large majorities of each group (See Table 3). Nearly all (94% of doctors and 97% of nurses and ward staff) supported mandatory testing for female sex workers (FSW), as well as for surgery patients (90% of doctors to 99% of nurses, *p<*0.001). Mandatory testing for surgery personnel was also endorsed by a majority, ranging from 73% of doctors, to 83% of nurses and to 88% of ward staff (*p<*0.001). Significantly more doctors (13%) than nurses and ward staff (both 5%, *p<*0.001) were in agreement with the statement that “health care workers should have the right to refuse to treat PLHIV.” The groups differed more widely in their endorsement of restricting PLHIV's rights to marry and have children. Slightly over half of the doctors did not think that HIV-positive women should be allowed to have children, compared to more than three quarters of both nurses and ward staff (*p<*0.001). Forty-one percent of doctors agreed that HIV-positive men should be denied the right to marry, as did 77% of nurses and 88% of ward staff (*p<*0.001). Similar proportions held the same opinion regarding HIV-positive women and marriage (37, 73 and 86%, respectively, *p<*0.001). There were no gender differences with respect to endorsement of coercive policies among any healthcare provider group.

**Table 3:** Endorsement of coercive policies and avoidance intentions towards PLHIV

	Doctors (*n=*305)		Nurses (*n=*369)		Ward staff (*n=*346)		
		
Individual items	%	*n*	%	*n*	%	*n*	*χ*^2^
Coercive policies							
Mandatory testing for FSW	94	287	97	358	97	337	5.38†
Mandatory testing for surgery patients	90	274	99	366	96	332	29.85***
Mandatory testing for surgery staff	73	223	83	305	88	302	22.27***
HIV-positive women banned from having children	55	168	76	279	80	275	53.98***
HIV-positive men should not be allowed to marry	41	124	77	283	88	306	186.16***
HIV-positive women not be allowed to marry	37	112	73	269	86	296	182.98***
HCW should not have to treat PLHIV	13	39	5	19	5	17	18.87***
Intent to discriminate: professional							
High likelihood of contact w/bodily fluids^a^	89	272	88	324	73	252	
Low likelihood of contact w/bodily fluids^b^	57	174	40	146	71	243	
Intent to discriminate: personal							
Change clinic or demand extra precautions if PLHIV were treated where you get care:	59	179	61	224	50	173	9.75**
Change school or avoid HIV-positive child if HIV-infected child in your child's school:	15	46	22	82	32	112	26.58***
Would not eat from plate used by PLHIV	42	128	53	193	56	195	13.62**
PLHIV should be treated in separate clinics	34	103	56	205	61	212	54.10***
Not comfortable feeding PLHIV by hand	33	98	27	101	21	72	11.04**
Not seek services from HIV-positive HCW	23	71	31	114	36	124	12.21**

a High likelihood of contact w/bodily fluids: doctors: examine open wound; nurses: draw blood; ward staff: change blood-stained linens of PLHIV; no between-group comparisons done due to different items.

b Low likelihood of contact w/bodily fluids: doctors: routine physical exam; nurses: dispense medication; ward staff: bathe PLHIV; no between-group comparisons done due to different items.

† *p<*0.10

* *p<*0.05

** *p<*0.01

*** *p<*0.001.

### Intent to discriminate

A large majority of participants responded that they would either refuse to perform, avoid physical contact or use more than standard precautions if they were asked to treat an HIV-positive patient (see Table 3). This included examining an open wound (89% of doctors), drawing blood (88% of nurses) or changing blood-stained linens (73% of ward staff). When asked about professional behaviours with a low risk of fluid contact, 57% of doctors stated that they would either refuse or take additional precautions before performing a routine physical examination. Similar responses were given by 40% of nurses before dispensing medication and 71% of ward staff before bathing a PLHIV. More than half of the doctors and ward staff and 39% of the nurses reported discriminatory intent in both situations. Only 10 to 19% of participants reported no intent to discriminate in any professional situation.

At least half of the participants in all subsamples said they would stop attending, or demand extra precautions if they were patients in clinics that served PLHIV. This proportion was higher among doctors (59%) and nurses (61%) than among ward staff (50%, *p<*0.01). But more ward staff (61%) and nurses (56%) than doctors (34%) agreed with the statement that PLHIV should be treated in separate clinics (*p<*0.001), and stated that they would not seek services from an HIV-positive healthcare provider (36, 31 and 23%, respectively; *p<*0.01). Gender differences were found only among ward staff participants, with 77% of male ward staff expressing intent to discriminate if they had to bathe an HIV-positive patient, vs. 65% of female ward staff (*p=*0.01). When asked what they would do if an HIV-infected child attended their child's school, somewhat fewer participants – 15% of doctors, 22% of nurses, and 32% of ward staff (*p<*0.001) – showed discriminatory intent. In line with results regarding misconceptions, about half of the participants stated that they would not eat from a plate used by a PLHIV and about a quarter would not feel comfortable feeding a PLHIV by hand. The former was most common among ward staff, the latter among doctors, with the proportion of nurses in between for both items (both *p<*0.01). On average, doctors endorsed fewer personal discrimination intentions (mean=2.1) than nurses (mean=2.7) and ward staff (mean=2.9, *p<*0.001).

### Drivers of stigma

Results from the bivariate and final multivariate regression models are presented in Tables 4–6. Table 4 shows that doctors with greater instrumental stigma at work (*β*=0.24, *p<*0.001) and who did not know a PLHIV personally (*β*=0.13, *p=*0.033) reported higher endorsement of coercive policies than did doctors with lower instrumental stigma and those with a personal acquaintance with PLHIV. Instrumental stigma at work was also significantly related to higher intent to discriminate in personal situations (*β*=0.19, *p=*0.001), as were higher levels of negative feelings towards PLHIV (*β*=0.13, *p=*0.019), blame (*β*=0.11, *p=*0.044), transmission misconceptions (*β*=0.36, *p<*0.001), perceived stigmatizing community norms (*β*=0.11, *p=*0.038), and less frequent professional contact with PLHIV (*β*=0.11, *p=*0.047). Those with less frequent professional contact also had higher odds of showing discriminatory behaviour while performing a routine medical examination of a PLHIV (AOR=1.35; 95% CI=1.08–1.70) or dressing an open wound of a PLHIV (AOR=1.94; 95% CI=1.32–2.98). More transmission misconceptions was also associated with higher odds of discrimination during a routine examination (AOR=1.51; 95% CI=1.03–2.32). Gender was not associated with drivers of stigma or intent to discriminate among doctors.

**Table 4:** Bivariate and multivariate associations with outcomes, for doctors

	Bivariate Pearson *r*	Multivariate^a^	

		*β*	sig.
*Outcome: endorsement of coercive policies*		(*n=*271, *R* ^2^=0.11)	
Younger age	0.09^‡^		
Higher income (log-transformed)	−0.12^†^		
More negative feelings towards PLHIV	0.12*		
More blame	0.17**		
More work-related instrumental stigma	0.26***	0.241	.000
More non-work instrumental stigma	0.07^‡^		
More transmission misconceptions (4 items)	0.13*	0.108	.071
Lower transmission knowledge (15 items)	0.13		
Less frequent professional contact w/PLHIV	0.14*		
Not knowing any PLHIV personally	0.13*	0.125	.033
More symbolic stigma	0.18**	0.120	.077
*Outcome: intent to discriminate, personal life*		(*n=*265; *R* ^2^=0.32)	
Younger age	0.08^‡^		
Higher income (log-transformed)	−0.12^†^		
More negative feelings towards PLHIV	0.22***	0.133	0.019
More blame	0.24***	0.110	0.044
More work-related instrumental stigma	0.27***	0.188	0.001
More transmission misconceptions (4 items)	0.40***	0.364	0.000
Lower transmission knowledge (15 items)	0.16**		
Less frequent professional contact w/PLHIV	0.17**	0.110	0.047
Not knowing any PLHIV personally	0.13*		
More symbolic stigma	0.25***	0.121	0.056
More stigmatizing perceived community norms	0.11*	0.113	0.038
	Pearson *r*	AOR	95% CI
	
*Outcome: intent to discriminate, professional: routine exam*		(*n=*268)	
Non-Hindu religion	0.12*		
Unmarried	0.09^‡^		
Younger age	0.17**		
Higher income (log-transformed)	−0.11^†^		
More negative feelings towards PLHIV	0.17**		
More work-related instrumental stigma	0.16**	1.28^†^	(0.96–1.71)
More non-work instrumental stigma	0.08^‡^		
More transmission misconceptions (4 items)	0.12*	1.51*	(1.03–2.32)
Lower transmission knowledge (15 items)	0.10^†^		
Less frequent professional contact w/PLHIV	0.11^†^	1.35*	(1.08–1.70)
More symbolic stigma	−0.14*		
More stigmatizing perceived community norms	−0.12*	0.64^†^	(0.37–1.07)
*Outcome: intent to discriminate, professional: open wound*		(*n=*270)	
Non-Hindu religion	0.08^‡^		
Higher income (log-transformed)	−0.07^‡^		
More negative feelings towards PLHIV	0.12*		
More blame	0.15*		
More work-related instrumental stigma	0.18**		
Lower transmission knowledge (15 items)	0.14*		
Less frequent professional contact w/PLHIV	0.17**	1.94**	(1.32–2.98)
Not knowing any PLHIV personally	0.14*	2.27^†^	(0.93–5.38)
More stigmatizing perceived community norms	−0.08^‡^		

Note: all models adjusted for site.

β standardized regression coefficient; AOR, adjusted odds ratio; CI, confidence interval.

a Multivariate regression: final model, obtained via backward elimination starting from all variables bivariately associated at *p<*0.25, until all *p<*0.10.

‡ *p<*0.25

† *p<*0.10

* *p<*0.05

** *p<*0.01

*** *p<*0.001.

The bivariate correlations and multivariate regression models in Table 5 show that nurses with lower levels of HIV transmission knowledge had significantly higher mean levels of endorsement of coercive policies (*β*=0.13, *p=*0.022), younger age (*β*=0.10, *p=*0.051), and higher mean levels of negative feelings towards PLHIV (*β*=0.10, *p=*0.050). Intent to discriminate in personal life was significantly related to nurses’ being non-Hindu (*β*=0.10, *p=*0.036), having higher levels of negative feelings towards PLHIV (*β*=0.12, *p=*0.013), of work and non-work instrumental stigma (work: *β*=0.23, *p*<0.001; non-work: *β*=0.11, *p=*0.029), and of perceived stigmatizing community norms (*β*=0.12, *p=*0.010). Finally, nurses with more misconceptions (*β*=0.20, *p<*0.001) and less transmission knowledge (*β*=0.19, *p<*0.001) also had significantly higher levels of discriminatory intent in personal situations. In both professional situations, nurses’ intent to discriminate was significantly related to higher levels of instrumental stigma at work (medication: AOR=1.37; 95% CI=1.08–1.73; draw blood: AOR=1.56; 95% CI=1.09–2.30), but the two outcomes varied in their relation to other correlates. Unmarried nurses (AOR=1.76; 95% CI=1.08–2.88) and those with lower household income (AOR=0.32; 95% CI=0.13–0.79) showed higher odds of discrimination than married nurses and nurses with higher income, respectively, when dispensing medication to PLHIV, while for the “draw blood” item, it was nurses with higher household income (AOR=4.33; 95% CI=1.21–16.12) and younger nurses (AOR=1.04; 95% CI=1.01–1.07) who were more likely to express discriminatory intent. Finally, more transmission misconceptions (AOR=1.69; 95% CI=1.27–2.26) and greater symbolic stigma (AOR=0.77; 95% CI=0.60–0.97) were associated with treating PLHIV differently when dispensing medication.

**Table 5:** Bivariate and multivariate associations with outcomes, for nurses

	Bivariate Pearson *r*	Multivariate^a^	

		*β*	sig.
*Outcome: endorsement of coercive policies*		(*n=*367; *R* ^2^=0.05)	
Younger age	0.08^‡^	0.101	.051
More negative feelings towards PLHIV	10^†^	0.104	.050
More work-related instrumental stigma	0.10^†^		
More transmission misconceptions (4 items)	0.12*	0.097	.074
Lower transmission knowledge (15 items)	0.15**	0.127	.022
*Outcome: intent to discriminate, personal life*		(*n=*362; *R* ^2^= 0.26)	
Non-Hindu religion	0.14**	0.101	.036
More negative feelings towards PLHIV	0.17***	0.119	.013
More work-related instrumental stigma	0.30***	0.234	.000
More non-work instrumental stigma	0.23***	0.112	.029
More transmission misconceptions (4 items)	0.32***	0.195	.000
Lower transmission knowledge (15 items)	0.29***	0.193	.000
Less frequent professional contact PLHIV	0.08^‡^		
More stigmatizing perceived community norms	0.11*	0.124	.010
	
	Pearson *r*	AOR	95% CI
	
*Outcome: intent to discriminate, professional: dispense medication*		(*n=*344)	
Non-Hindu religion	0.08^‡^		
Unmarried	0.16**	1.76*	(1.08–2.88)
Younger age	0.09^†^		
Higher income (log-transformed)	−0.14**	0.32*	(0.13–0.79)
More negative feelings towards PLHIV	0.09^†^		
More work-related instrumental stigma	0.15**	1.37**	(1.08–1.73)
More non-work instrumental stigma	0.10^†^		
More transmission misconceptions (4 items)	0.22***	1.69***	(1.27–2.26)
Lower transmission knowledge (15 items)	0.13*		
Less frequent professional contact PLHIV	0.11*		
More symbolic stigma	−0.09^†^	0.77*	(0.60–0.97)
*Outcome: intent to discriminate, professional: draw blood*		(*n=*359)	
Younger age	0.16**	1.04*	(1.01–1.07)
Higher income (log-transformed)	0.11*	4.33*	(1.21–16.12)
More negative feelings towards PLHIV	0.13*	1.01^†^	(1.00–1.03)
More work-related instrumental stigma	0.19***	1.56*	(1.09–2.30)
More transmission misconceptions (4 items)	0.07^†^		
Not knowing any PLHIV personally	−0.10^†^	0.47^†^	(0.18–1.07)

Note: all models adjusted for site.

*β*, standardized regression coefficient; AOR, adjusted odds ratio; CI, confidence interval.

a Multivariate regression: final model, obtained via backward elimination starting from all variables bivariately associated at *p*<0.25, until all *p*<0.10.

‡ *p<*0.25

† *p<*0.10

* *p<*0.05

** *p<*0.01

*** *p<*0.001.

The bivariate correlations and multivariate regression models for ward staff are shown in Table 6. Endorsement of coercive policies and intent to discriminate in personal situations were both significantly related to more negative feelings towards PLHIV (*β*=0.15, *p=*0.006; *β*=0.15, *p=*0.003, respectively), more blame (*β*=0.13, *p=*0.020; *β*=0.12, *p=*0.013), more misconceptions (*β*=0.13, *p=*0.014; *β*=0.34, *p<*0.001) and more symbolic stigma (*β*=0.14, *p=*0.014; *β*=0.14, *p=*0.006). In addition, intent to discriminate in personal situations also increased with younger age (*β*=0.17, *p<*0.001), decreasing frequency of professional contact with PLHIV (*β*=0.11, *p=*0.025), and increasing levels of instrumental stigma at work (*β*=0.22, *p<*0.001). Male ward staff were twice as likely (AOR=1.98; 95% CI=1.21–3.28) to discriminate when bathing patients than female ward staff. Males were also more likely than females (AOR=1.95; 95% CI=1.13–3.45) to discriminate when asked to change a PLHIV's blood-stained linens. Having more negative feelings towards PLHIV (AOR=1.10; 95% CI=1.00–1.02), greater work-related instrumental stigma (AOR=1.66; 95% CI=1.28–2.19) and less frequent professional contact with PLHIV (AOR=1.22; 95% CI=1.00–1.49) were also associated with higher odds of discrimination in this situation.

**Table 6:** Bivariate and multivariate associations with outcomes, for ward staff

	Bivariate Pearson *r*	Multivariate^a^	

		*β*	sig.
*Outcome: endorsement of coercive policies*		(*n=*318; *R* ^2^=0.14)	
Non-Hindu religion	0.12*		
More negative feelings towards PLHIV	0.21***	0.151	0.006
More blame	0.20***	0.126	0.020
More work-related instrumental stigma	0.22***	0.111	0.073
More non-work instrumental stigma	0.12*		
More transmission misconceptions (4 items)	0.20***	0.134	0.014
Less frequent professional contact w/PLHIV	0.15**		
More symbolic stigma	0.12*	0.143	0.014
*Outcome: intent to discriminate, personal life*		(*n=*314; *R* ^2^=0.37)	
Younger age	0.13*	0.169	0.000
More negative feelings towards PLHIV	0.24***	0.145	0.003
More blame	0.22***	0.120	0.013
More work-related instrumental stigma	0.30***	0.216	0.000
More non-work instrumental stigma	0.18***		
More transmission misconceptions (4 items)	0.42***	0.335	0.000
Lower transmission knowledge (15 items)	0.24***	0.091	0.072
Less frequent professional contact w/PLHIV	0.22***	0.107	0.025
More symbolic stigma	0.20***	0.140	0.006
More stigmatizing perceived community norms	0.14**		
	
	Pearson *r*	AOR	95% CI
	
*Outcome: intent to discriminate, professional: bathe PLHIV*		(*n=*336)	
Male gender	0.14*	1.98**	(1.21–3.28)
Unmarried	0.06^‡^		
Younger age	0.09^†^		
Not knowing any PLHIV personally	−0.08^‡^		
More stigmatizing perceived community norms	−0.08^‡^	1.59^†^	(0.97–2.62)
*Outcome: intent to discriminate, professional: change blood-stained linens*		(n=314)	
Male gender	0.06^‡^	1.95*	(1.13–3.45)
Younger age	0.06^‡^		
Lower education	−0.06^‡^		
More negative feelings towards PLHIV	0.14*	1.10**	(1.00–1.02)
More work-related instrumental stigma	0.15**	1.66***	(1.28–2.19)
More non-work instrumental stigma	0.09^†^		
More transmission misconceptions (4 items)	0.10^†^		
Less frequent professional contact w/PLHIV	0.10^†^	1.22*	(1.00–1.49)
Not knowing any PLHIV personally	−0.06^‡^	0.57^†^	(0.29–1.05)
More symbolic stigma	0.11*		
More stigmatizing perceived community norms	0.06^‡^		

Note: all models adjusted for site.

β standardized regression coefficient; AOR, adjusted odds ratio; CI, confidence interval.

a Multivariate regression: final model, obtained via backward elimination starting from all variables bivariately associated at *p<*0.25, until all *p<*0.10.

‡ *p<*0.25

† *p<*0.10

* *p<*0.05

** *p*<0.01

*** *p*<0.001.

## Discussion

The results reveal disturbingly high rates of stigma attitudes and intent to discriminate among doctors, nurses and ward staff in these urban healthcare settings. The rates are similar to those reported by outpatients in these settings as well as to results of studies conducted in other parts of the country [11–13, 30, 32–36, 38, 42] and may thus represent wider community norms. The almost universal endorsement of mandatory testing for FSW and surgery patients may be one of the reasons why testing is now routinely performed in Indian healthcare settings for surgery patients and pregnant women. Routine periodic testing of key populations is also currently done in some areas. Although doctors were less likely (37 to 55%) than nurses (73 to 76%) and ward staff (80 to 88%) to endorse the different coercive measures in relation to marriage and children, their rates were still surprisingly high. These endorsements are of particular concern since they involve the denial of basic human rights of PLHIV to enjoy marital status and parenthood, which are crucial aspects of Indian culture. These findings highlight the need for a rights-based approach to addressing stigma in future regional and national intervention programmes.

Participants also reported high rates of intent to treat HIV-positive patients differently from uninfected patients, both in situations that involved a risk of fluid exposure and in situations that are typically considered low risk. Since female ward staff reported more transmission misconceptions and a more negative view of PLHIV, the finding that male ward staff were more likely to report intent to discriminate may reflect their perception that they have more control over their job duties than their female counterparts. This needs to be explored further to determine how to best address this gender difference in a stigma reduction intervention. It was encouraging that physicians and nurses were significantly less likely to state that they intended to discriminate in low-risk situations; however, health care professionals who use universal precautions do not need to use double gloves or avoid HIV-infected patients in order to be safe. In addition to stigma, these high rates might also be indicative of lack of confidence in standard universal measures to prevent infection.

Intent to discriminate was only slightly less in non-professional situations. The majority of all groups stated that they did not want to be treated in the same clinics as PLHIV and more than half of the nurses and ward staff reported that they would be unwilling to eat from the same plate as an infected individual. This item was endorsed by 42% of the doctors also.

Although there are minor variations, the drivers of stigma and discrimination appear to be fairly consistent across the different groups. Transmission-related fears and misconceptions, as well as limited experience working with PLHIV, blame and negative feelings towards PLHIV seem to be driving both endorsement of coercive measures and intent to discriminate against PLHIV in personal and professional contexts, regardless of whether the latter situations actually involve risk of fluid exposure. This is consistent with findings from previous studies [30, 31, 33, 34], and our previous paper on stigma among outpatients in Mumbai and Bengaluru [38], suggesting that misconceptions are a consistent driver of HIV stigma in India. The findings from this study thus indicate that stigma reduction interventions need to target common misconceptions, even among highly educated and already trained healthcare providers. Since younger and less experienced nurses and ward staff were more likely to discriminate, there may also be a need to ensure that they are thoroughly trained in universal precautions until they are comfortable and confident in their ability to prevent transmission.

The fact that more experience with PLHIV was associated with lower rates of stigma and discrimination in all three groups suggests that interventions may be more effective if PLHIV are involved at all stages of intervention development and implementation to ensure sufficient and meaningful interactions. It might also be helpful to involve experienced healthcare providers, who have extensive experience treating PLHIV as role models for their junior colleagues to provide opportunities for observational learning, help change norms in the workplace and to increase the likelihood of intervention sustainability. Doctors treating PLHIV respectfully are also likely to make an impression and set a standard for both nurses and ward staff in their institutions, given the hierarchical nature of relationships in these settings.

Both female doctors and female ward staff reported a greater number of transmission misconceptions than did their male counterparts, in spite of their very different levels of education. This suggests that there may be differences in HIV-related education received by male and female students in Indian schools. It is thus important for future HIV prevention and stigma interventions to address basic transmission facts when targeting female participants, regardless of their level of education.

Similar to every study, ours has a number of limitations that need to be considered when interpreting its results. Since this study used a cross-sectional design, we are unable to draw conclusions about causality and can only state which variables are associated. Future research is needed to examine these relationships in a longitudinal fashion to clarify the nature of these associations. In addition, the generalizability of these findings is limited to the types of healthcare settings that collaborated with us in these two large urban areas. We made every effort to recruit healthcare providers from a wide range of clinics and hospitals, in order to be as representative as possible of healthcare settings that are accessible to patients of all socioeconomic backgrounds. However, our sample did not include healthcare providers in non-allopathic institutions. We are also limited by our reliance on self-reported measures, which may be subject to social desirability biases. Additional studies using behavioural observations are needed to provide data on enacted stigma in these settings.

## Conclusions

The high rates of stigma and discrimination among healthcare providers in these urban Indian healthcare settings appear to be driven primarily by negative feelings towards PLHIV, lack of experience as well as misconceptions and fear of casual transmission. Stigma reduction interventions are thus urgently needed to target transmission misconceptions and to increase interactions with PLHIV. Such programmes need to be designed and implemented in collaboration with PLHIV networks and use a rights-based and gender-sensitive approach. In order to be both effective and sustainable, interventions should ideally make use of professional role models and be integrated into existing training structures in hospital clinics and the curricula in nursing and medical schools.
